# Laparoscopic Surgical Options as a Minimally Invasive Procedure for a Patient With Recurrent Postoperative Pain in Anterior Cutaneous Nerve Entrapment Syndrome: A Case Report

**DOI:** 10.7759/cureus.39366

**Published:** 2023-05-23

**Authors:** Yoshitaka Kondo, Toshiyoshi Fujiwara

**Affiliations:** 1 Department of Gastroenterological Surgery, Okayama University Graduate School of Medicine, Dentistry, and Pharmaceutical Sciences, Okayama, JPN

**Keywords:** anterior cutaneous nerve entrapment syndrome, low pain threshold, intraperitoneal approach, laparoscopic neurectomy, refractory abdominal pain, acnes

## Abstract

This report presents a case of a 70-year-old woman who developed anterior cutaneous nerve entrapment syndrome (ACNES) three years ago and had an anterior cutaneous neurectomy in the left Th10 region. Postoperatively, the pain had improved entirely, but 10 weeks later, she developed a recurrence in the vicinity of the wound. The anterior intercostal nerve branch (Th10), located between the transversus abdominis and internal oblique muscles, was dissected laparoscopically six months after the initial surgery. There was no re-recurrence of pain for four months postoperatively. The postoperative recurrence of ACNES was refractory to various treatments, including surgical neurectomy, and is often difficult to treat. In cases in which transversus abdominis plane block is effective, laparoscopic neurectomy through an intraperitoneal approach may be effective, and minimally invasive laparoscopic treatment may be an effective surgical option for patients with recurrent and refractory ACNES who have a low pain threshold and are prone to prolonged complaints due to wound pain.

## Introduction

Anterior cutaneous nerve entrapment syndrome (ACNES) is a chronic abdominal wall pain caused by entrapped intercostal nerve as it passes through the anterior sheath of the abdominal rectus muscle [1]. Anterior neurectomy is effective for patients who do not experience long-term relief from treatment with local anesthetic injections [2]. However, treatment for patients for whom neurectomy is ineffective is often challenging. For recurrent patients for whom local blockade is effective, a neurectomy with consideration of dissection up to the posterior sheath of the abdominal rectus muscle has been reported to be effective [3]. However, for cases where a neurectomy has already been performed up to the level of the posterior sheath, there are few options for effective treatment. Herein, we report a case of successful treatment for a refractory recurrent pain through laparoscopic neurectomy at the level of the transversus abdominis muscle.

## Case presentation

A 70-year-old Japanese woman had been experiencing needle-like tingling pain in the left lower abdomen for the past three years. Her occupation was an office worker. She had no underlying disease and no history of hospitalization or surgery. She also had no evidence of trauma, medication, or other factors that could be risk factors for ACNES. At first, she could tolerate the pain in her lower left abdomen, but gradually she began to experience excruciating pain. Her pain fluctuated diurnally and worsened when she worked long hours at her desk. It was difficult for her to work until regular hours during busy periods. Prescribed analgesic agents had little effect, and she had to rest and wait for the pain to pass. She gradually became unable to go jogging and became increasingly depressed. She visited a local hospital and underwent upper and lower intestinal endoscopy and computed tomography, but no abnormality was found. She visited our hospital's department of general medicine and was diagnosed with ACNES at her first visit based on physical findings, including Carnett's sign and the effect of trigger point injections. Although her pain disappeared after a trigger point injection, the pain flared up three days after local blocks. Systemic drug administration, such as nonsteroidal anti-inflammatory drugs, antiepileptics, antidepressants, and weak opioids, was ineffective. Under local anesthesia, an anterior cutaneous neurectomy was performed on the left Th10. Pain intensity was evaluated using the Numeric Rating Scale (NRS). Upon initial assessment at her first visit to our department, the patient's NRS score was recorded as nine. Postoperatively, a significant decrease in the NRS score to zero was observed, indicating the absence of pain remained resolved at eight weeks of outpatient follow-up. Given the NRS score of zero postoperatively, administering analgesics was deemed unnecessary during the postoperative period. At 10 weeks postoperatively, pain flared up in line with the wound. The NRS score had escalated to eight at the recurrence time. No discernible triggers for these pain flare-ups could be identified. Local trigger points on the anterior or posterior sheath of the abdominal rectus muscle were ineffective. After confirming temporary pain relief with the transversus abdominis plane (TAP) block in the left Th10 region, the anterior intercostal nerve branch (Th10), located between the transversus abdominis and internal oblique muscles, was dissected laparoscopically six months after the initial surgery. The ACNES pain disappeared the day after the operation, and the wound pain was almost entirely resolved within a week. Four months postoperatively, there was no recurrence of pain.

Surgical procedure

**Figure 1:**
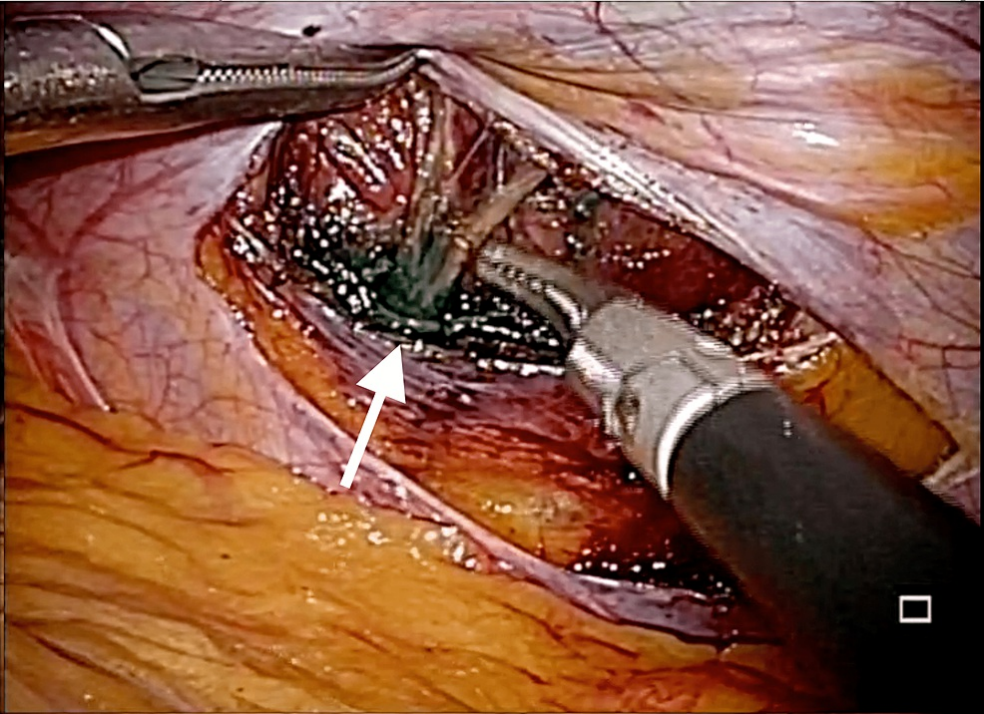
The intraoperative view of the intercostal nerve. The anterior intercostal nerve branch (Th10) between the transverse abdominal muscle and the internal abdominal oblique muscle was marked with indocyanine green.

Ultrasound-guided pulse stimulation was attempted to induce target pain in the transverse abdominal muscle on the median side of the tender point. This nerve was determined to be responsible and was blocked by marking 1 ml of 2% xylocaine mixed with indocyanine green (ICG) around this nerve. The NRS score was observed to decrease to zero or one within five minutes following the implementation of the block. General anesthesia was introduced after confirming that the TAP block had eliminated the pain. The 5 mm trocars for an endoscope were inserted through the umbilicus. Two 5 mm ports were placed on the contralateral abdominal wall of the upper and lower abdomen, respectively, to begin the procedure. The intra-abdominal insufflation pressure was meticulously regulated at 8 mmHg to minimize potential irritation to the abdominal wall. An ICG was observed through the peritoneum. A 3-cm incision was made perpendicular to the peritoneal nerve pathway. The transversus abdominis muscle was split along the muscle fibers to reach the dorsal surface of the internal oblique abdominal muscle. The nerve running between the transversus abdominis and internal oblique muscle was identified using a dye as a marker (Figure 1). The nerve was ligated and excised at a width of approximately 1 cm. Ideally, the nerve should be transected without ligation or clipping, while the accompanying vasculature should be ligated or clipped. Nonetheless, in the present case, the intricacy of vessel-nerve separation necessitated ligation. The peritoneum was repaired with absorbable thread, and the abdomen was closed to complete the procedure.

## Discussion

In this report, we describe a case in which a strategy of securely conducting a more central nerve transection via laparoscopy proved effective for treating ACNES recurrence, even when the responsible nerve had initially been transected. For cases of refractory recurrent ACNES post-surgery, where treatment options are limited, it is theoretically plausible that identifying, marking, and then transecting the nerve more centrally than the initial neurectomy site via laparoscopy could be effective. While there are reports of nerve transections performed at the level of the posterior sheath in recurrent cases [3], instances documenting the laparoscopic transection of a more central portion of the nerve are rare.

Anterior neurectomy for ACNES appears successful in approximately 70% of operated patients [2]. However, treatment for cases where neurectomy is ineffective, or cases of recurrence can often be complex. The option of re-resection for recurrent cases was reported as effective [3]. If the pain was due to previously unresected end twigs, persistent neurovascular branches found at the level of the anterior sheath of the abdominal rectus muscle will be removed. If the secondary anterior neurectomy revealed no residual end twigs, the neurectomy at the level of the posterior sheath of the abdominal rectus muscle will be performed. The choice of surgical technique depends on the preoperative local block and physical examination, as well as the scar tissue and condition of the nerve stump during surgery.

In cases where the patient initially received a relatively extensive nerve resection up to the partial abdominal rectus muscle and posterior sheath, including the anterior sheath, and there was little effect from the preoperative posterior block, there may be little surgical efficacy with neurectomy only up to the posterior sheath.

A local block is a temporary and chemical nerve blockade. It seems reasonable to perform a neurectomy at the position where the local block is most effective. For patients with an effective TAP block, a neurectomy at the more central part of the intercostal nerves above the transverse abdominal muscle may be effective [4].

Upon reflection, an initial laparoscopic central nerve transection could have obviated the need for subsequent surgery. However, the etiology of ACNES is fundamentally attributed to the nerve foramen, which is constricted by the anterior sheath [1]. Even if an initial central nerve transection was performed, persistent pain stimuli from the area of responsibility, coupled with nerve regeneration, elevate the risk of recurrence. Consequently, the primary surgical approach should focus on anterior nerve transection rather than laparoscopy [3].

Furthermore, in patients with ACNES, the pain threshold is often decreased. Therefore, the pain from the surgical incision may be more potent than that of patients with equivalent incisions without ACNES [5,6]. Even if the pain from ACNES improves, there is a risk of prolonged pain from the surgical incision in general. Although no prolonged wound pain was observed in this case, the author has encountered similar cases where wound pain persisted for up to three months after the resolution of ACNES itself. Therefore, minimizing the surgical incision through an intra-abdominal approach to the nerves using laparoscopy is rational and practical for patients with ACNES.

In addition, even if lateral cutaneous nerve entrapment syndrome is present as a postoperative condition, neurectomy on the transverse abdominal muscle may be effective, making it a versatile surgical technique for various indications.

We anticipate that the treatment will be considerably challenging for re-recurrence cases. There is no reported effective treatment in case of recurrence following multiple surgeries. Post-laparoscopic treatment involves reverting to the fundamental treatment of ACNES, initially involving conservative treatment with systemic oral medications, followed by nerve blocks or pulse ablation therapy. If the efficacy of the more central nerve blocks can be confirmed, we may consider severance at the higher-level nerves. However, theoretically, for recurrence after having undergone nerve severance surgery twice - which should ideally have been curative - there is a high probability that a third surgery merely severing the nerves would not lead to remission. If surgical excision becomes the only viable option, it would be necessary to thoroughly investigate the source of the pain and tailor our approach to each case. For example, we may consider a terminal excision for neuroma at the nerve endings, whereas scar excision may be appropriate for pain due to scar tissue.

The present study's limitations lie in the reliance on the subjective evaluation of a single patient, which undermines the robustness and generalizability of the results. Therefore, there is a compelling need for a larger study to address these deficiencies. Notwithstanding these limitations, the reported success of complete posterior nerve transection in recurrent cases offers theoretical justification for pre-blocking the nerve [3], identifying it through dye-assisted visualization, and proceeding with transection. Furthermore, the application of a minimally invasive laparoscopic approach, characterized by smaller wound size, appears to be a reasonable strategy, particularly for ACNES patients exhibiting a low pain threshold.

## Conclusions

We experienced a case of postoperative recurrence of ACNES in a patient who had benefited from laparoscopic neurectomy over the transverse abdominal muscle as a minimally invasive surgery. A less invasive laparoscopic procedure is reasonable for patients with a lowered pain threshold. When pain is localized, and local blocks of the target nerve are effective, laparoscopic neurectomy to the level of the transversus abdominis muscle may be a new treatment option for patients with refractory recurrence after nerve transection.

## References

[1] Boelens OB, Scheltinga MR, Houterman S, Roumen RM (2011). Management of anterior cutaneous nerve entrapment syndrome in a cohort of 139 patients. Ann Surg.

[2] Boelens OB, van Assen T, Houterman S, Scheltinga MR, Roumen RM (2013). A double-blind, randomized, controlled trial on surgery for chronic abdominal pain due to anterior cutaneous nerve entrapment syndrome. Ann Surg.

[3] van Assen T, Boelens OB, van Eerten PV, Scheltinga MR, Roumen RM (2014). Surgical options after a failed neurectomy in anterior cutaneous nerve entrapment syndrome. World J Surg.

[4] Yoshitaka K (2019). Sudden abdominal pain leading to chronic abdominal pain. J Generalist Med.

[5] van Rijckevorsel DC, Boelens OB, Roumen RM, Wilder-Smith OH, van Goor H (2017). Treatment response and central pain processing in anterior cutaneous nerve entrapment syndrome: an explorative study. Scand J Pain.

[6] Tsuchida T, Kondo Y, Ishizuka K, Matsuda T, Ohira Y (2022). Nerve identification procedures are necessary for complete recovery from recurrent cases of anterior cutaneous nerve entrapment syndrome: a case report. Cureus.

